# Endocardial-to-mesenchymal transition underlies cardiac outflow tract septation and bicuspid aortic valve formation in the Syrian hamster model

**DOI:** 10.1038/s41598-025-91454-6

**Published:** 2025-03-12

**Authors:** María Teresa Soto-Navarrete, Bárbara Pozo-Vilumbrales, Miguel Á. López-Unzu, Laura Martín-Chaves, Ana C. Durán, Borja Fernández

**Affiliations:** 1 Department of Animal Biology, Faculty of Science, University of Malaga, Malaga, Spain; 2 Biomedical Research Institute of Malaga and Nanomedicine Platform-IBIMA BIONAND Platform, Malaga, Spain; 3 Spanish National Centre for Cardiovascular Research (CNIC), Madrid, Spain; 4 Heart Area, Virgen de la Victoria University Hospital, Malaga, Spain; 5 Center for Biomedical Research Network - Cardiovascular Diseases (CIBERCV), Malaga, Spain

**Keywords:** Bicuspid aortic valve, Cardiac outflow tract, Endocardial cushion fusion, Endocardial-mesenchymal transition, Hamster embryo, Cardiovascular models, Animal disease models, Confocal microscopy, Hamster, Congenital heart defects, Valvular disease, Heart development, Epithelial-mesenchymal transition, Embryology, Immunohistochemistry

## Abstract

Right-left bicuspid aortic valve (R-L BAV) is the most frequent phenotype of the most common congenital heart disease. Its etiology is based on two associated morphogenetic defects during cardiac outflow tract (OFT) septation: abnormal migration of cardiac neural crest (CNC) cells, and excessive fusion of the conal ridges (CRs). The aim of this study is to elucidate the mechanism involved in the fusion of the CRs responsible for normal and abnormal OFT septation and BAV formation. Two mechanisms have been proposed: endocardial apoptosis and endocardial-mesenchymal transition (EMT). The involvement of these mechanisms in the fusion event was tested in embryos of the hamster model with BAV. Apoptotic cells were absent in the fusion area of the CRs. However, we detected endocardial cells (CD34^+^;VE-Cadherin^+^) showing positive signals for migration markers (α-actin^+^) in the fusion area of the CRs of embryos developing both normal aortic valve and BAV. These cells showed an intermediate morphological phenotype between endocardial and mesenchymal cells. The findings clearly indicate that EMT, and not apoptosis, is the cellular mechanism underlying the normal and excessive fusion of CRs that give rise to tricuspid aortic valve and BAV, respectively. Furthermore, our results show that the fusion of CRs in embryos developing BAV continues after the OFT septation, suggesting over-induction of EMT by abnormally distributed CNC cells.

**Supplementary Information:**

The online version contains supplementary material available at 10.1038/s41598-025-91454-6.

## Introduction

**Fig. 1:**
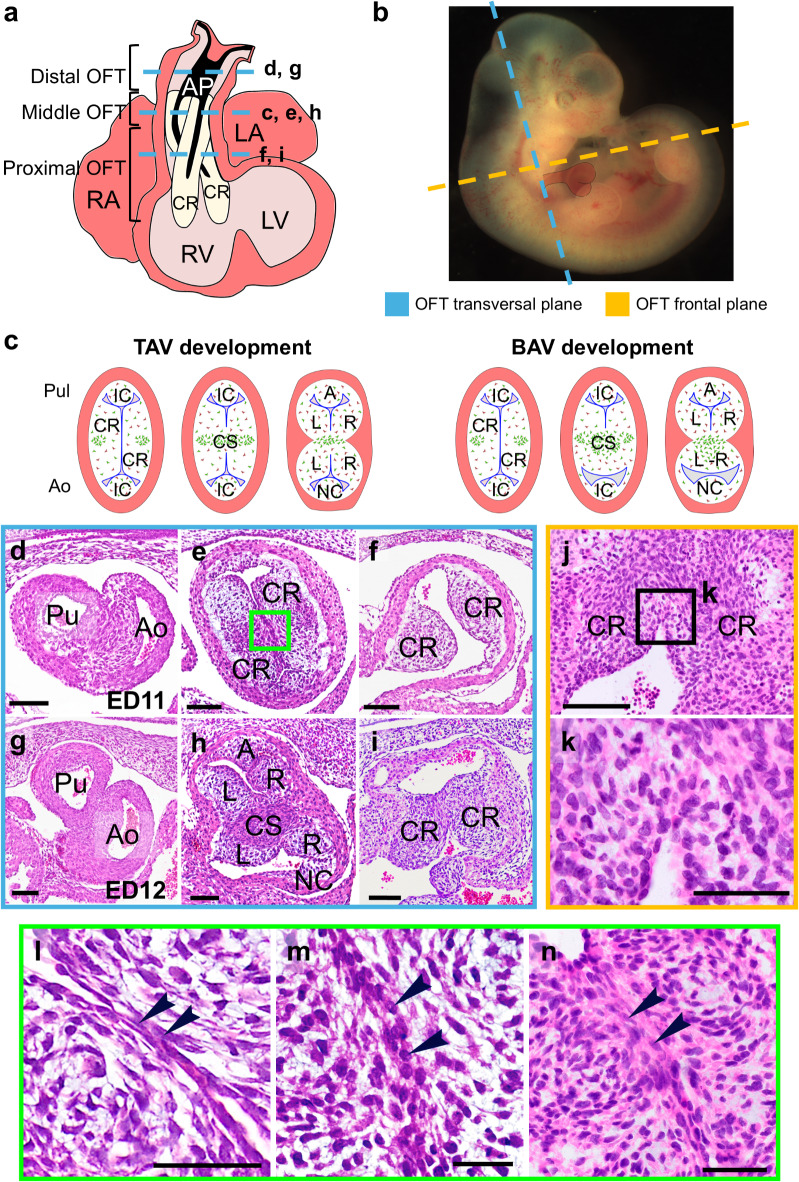
(**a-c**) Schematic representations of an embryonic heart in frontal (**a**), anatomical position (**b**) and transverse (**c**) views during outflow tract (OFT) septation. The frontal view (**a**) shows conal ridges (CRs) along the OFT, and the distribution of the aortic-pulmonary septation complex (AP) that shows an inverted-U shape (in black), composed of cardiac neural crest (CNC) cells. The schematic representation in (**b**) shows the orientation of the frontal and transversal sections of the OFT used in the study with respect to a hamster embryo. The transverse views (**c**) represent consecutive stages of the septation of tricuspid (TAV) and bicuspid aortic valves (BAV). During the normal fusion of the conal ridges (CR), CNC cells (in green), initially distributed in prongs, migrate to the central portion to form the conal septum (CS). A CNC abnormal migration extends the fusion of the posterior margins of the PR and SR in BAV embryos. The CS divides the *conus* in two aortic (Ao) and pulmonary (Pul) independent OFTs, each containing three (TAV) or two (BAV) valve cushions. (**d**-**k**) Transverse (**d**-**f**,** g**-**i**, ) and frontal (**j**,**k**) sections of ED11 (**d**-**f**,**j**,**k**) and ED12 (**g**-**i**) embryonic hearts show the OFT septation in the Syrian hamster. Hematoxylin-eosin. At ED11, the distal OFT (**d**) shows a mesenchymal appeareance, whereas proximally, at the *conus* (**e**) the fusion of CRs occurs at the location of the prospective semilunar valves (**e**,**j**), but not in the most proximal region (**f**). At ED12, the distal OFT (**g**) already shows an arterial appearance, whereas proximally, the CS is formed between the semilunar valves primordia (**h**), and the fusion reaches the most proximal region of the OFT (**i**). Green (**e**) and black (**j**) squares show magnifications of fusion area in consecutive transverse sections (**l**-**n**) and frontal sections (**k**), respectively. The central portion of the two opposite CRs get in contact (**l**) and fuse (**k**,**m**,**n**). Arrowheads indicate endocardial cell in contact (**l**), endocardial/mesenchymal cells (**m**) and transformed (mesenchymal) cells (**n**) in consecutive sections of the area of fusion. A: anterior intercalated cushion; L: left aortic or pulmonary valve cushion; LA: left atrium; LV: left ventricle; R: right aortic or pulmonary valve cushion; RA: right atrium; RV: right ventricle. Scale bars for insets: 100 μm. Scale bars for magnifications: 50 μm.

The embryonic cardiac outflow tract (OFT) is the most cranial segment of the embryonic heart. Initially, it consists of a single tubular myocardial chamber, the *conus*, that connects the right ventricle with the aortic sac and the aortic arch arteries. At approximately human Carnegie stage 15^1^, extra-pericardial second heart field (SHF) and cardiac neural crest (CNC) cells enter the pericardial cavity, forming the distal component of the OFT formerly referred as *truncus*, i.e., the arterial pole of the heart (Fig. 1a,b,d). Proximally, the *conus* (middle and proximal components of the OFT) contains two long, helical, and opposing mesenchymal cushions covered by endocardium named conal ridges (CRs) (Fig. 1a,b,e,f). CRs are involved in the process of septation of the OFT and their distal portions, corresponding to the middle OFT, form the primordia of the semilunar valves together with two smaller mesenchymal cushions called intercalated cushions (IC), intercalated ridges or intercalated valve swellings (Fig. 1c,e,h). OFT septation starts with a protrusion from the dorsal wall of the aortic sac that grows into the distal OFT forming the aortic-pulmonary septum (Fig. 1a,d,g). When the aortic-pulmonary septum reaches the middle OFT, conal septation takes place through the formation of the conal septum (CS), after the fusion of the two facing CRs (Fig. 1a,c,h)^2–7^. This fusion event occurs when the endocardium lining the two opposing and contacting CRs disappears, allowing cellular continuity between the CRs, resulting in a unique mesenchymal mass (Fig. 1c,e,j,k). Then, CNC-derived mesenchymal cells aggregate into this mesenchymal mass to form the CS (Fig. 1c)^2,3,6,8^. Besides the well-known role of CNC cells in conal septation, and the numerous genes that have been shown to be involved in the process (reviewed in^9^), the cellular mechanism underlying the fusion of CRs remains unknown.

The margins of the CRs constitute the primordia of the right and left semilunar valve leaflets and sinuses, the so-called right and left aortic and pulmonary valve cushions (Fig. 1c,h). The ICs, located anteriorly and posteriorly in the OFT (Fig. 1c,h), form the primordia of the posterior (non-coronary) and anterior leaflets and sinuses of the aortic and pulmonary valves, respectively^7,10^. As a result of septation, six mesenchymal cushions arise in the OFT: the right and left aortic and pulmonary valve cushions, derived from the CRs, and the anterior pulmonary and posterior or non-coronary aortic valve cushions, derived from the ICs (Fig. 1c,h). The aortic valve cushions acquire the adult semilunar morphology through a still poorly understood process called excavation^11–13^.

Bicuspid aortic valve (BAV) is the most common congenital heart disease in humans, with an incidence of 1–2% in the general population^14,15^. The anatomical elements that comprise a normal or tricuspid aortic valve (TAV) are reduced in BAVs, and two anatomical morphotypes have been described. The antero-posterior BAV, also called right-left (R-L) or BAV type A, consists of two sinuses and leaflets arranged in antero-posterior orientation. In contrast, the latero-lateral BAVs (BAV type B or right-non-coronary -R-NC-, and BAV type C or left-noncoronary -L-NC-) also show two sinuses and leaflets but located at the right and left side of the aortic root^16,17^. R-L BAV is the most frequent BAV morphotype (70–80%), followed by R-NC BAV (20–30%) and L-NC BAV (3–6%)^17–19^.

The R-L BAV results from a defect during the OFT septation process: the excessive fusion of the CRs accompanied by an altered organization of the CS^9,20^ (Fig. 1c). As a result of the septation defect, the right and left aortic valve cushions develop as fused structures. CNC cells are involved in both endocardial cushion formation and conal septation^2,3,8,21^. These cells, which come from the neural tube, migrate along the OFT to participate in the correct formation and positioning of CRs and ICs. Subsequently, they induce and regulate the conal septation process, forming the CS. Although the participation of CNC cells in the OFT septation is well established, the cause of the extra-fusion of CRs is not understood in detail due to the lack of knowledge of the cellular mechanism involved in the normal process of fusion of the CRs.

Currently, only one spontaneous animal model of non-syndromic BAV has been described. It consists of an inbred strain (T strain) of Syrian hamster (*Mesocricetus auratus*) with a relatively high (~40%) incidence of R-L BAV^9,13,20,22–25^. This animal model has substantially contributed to the current knowledge on BAV etiology^9,13,14,17,20,22,26^. The anatomy and inheritance of BAV in the hamster model are similar to those in patients^23,24^.

The aim of this study was to elucidate the cellular mechanism involved in the fusion of the CRs of hamster embryos from the T strain and a control strain with null incidence of BAV. Three fusion mechanisms have been proposed to operate during embryonic development: epithelial adhesion, epithelial apoptosis and epithelial-mesenchymal transition (EMT)^27^. Apoptosis and EMT were selected to be tested as possible candidate mechanisms. For this purpose, we examined hamster embryos at the stage of conal septation (embryonic day (ED) 11 and ED12) from the T and the control strain, and assessed several experiments, looking for evidence of apoptosis and EMT in the fusing CRs. Apoptosis was assessed by the TUNEL assay and immunofluorescence for active caspase 3. EMT was assessed by cellular morphology in semithin sections and double immunofluorescence for markers for endocardium (CD34 and VE-Cadherin) and cell migration (α-actin).

## Results

### Normal conal septation process in the Syrian hamster

Figure 1 shows the conal septation process in transverse (d-i, l-n) and frontal (j, k) sections of the OFT of Syrian hamster embryos. The conal septation occurs between ED11 and ED12 (Fig. 1d-i). At ED11 stage, the embryonic cardiac OFT is a myocardial tube containing four cushions (Fig. 1c,e). The CRs are two long, helical, and opposite endocardial cushions extending through the entire OFT, named septal and parietal CRs. The other two cushions, the anterior and posterior ICs are smaller and restricted to the medial region of the OFT (distal *conus*), at the level where the semilunar valves are formed. The CRs and the ICs are composed of mesenchymal cells lined by a layer of endocardial cells. The conal septation process begins when the central portion of the CRs get fused, which takes place disto-proximally along the *conus* (Fig. 1d-i). At ED11 (Fig. 1d-f), after the formation of the aortic-pulmonary septum in the most distal portion of the OFT (Fig. 1d), the fusion of the CRs starts in the middle portion, i.e., the prospective location of the semilunar valve primordia (Fig. 1e), still not affecting the proximal portion of the OFT (Fig. 1f). At ED12 (Fig. 1g-i), a mesenchymal condensation is formed at the middle-region of the OFT, corresponding to the CS (Fig. 1h). At this stage, the fusion reaches the most proximal region of the OFT (Fig. 1i). The detailed inspection of the fusion of the CRs at ED11 and ED12 showed that it starts with the contact of the endocardial linings covering the opposite CRs (Fig. 1l), followed by the disappearance of the endocardial cells in the center of the ridges (Fig. 1e,i,j-n). The region of the CRs where the contacting endocardial cells disappear delimits the area of fusion. The CS divides the single OFT into two independent OFTs, each containing three mesenchymal cushions, the valve cushions, which form the aortic and pulmonary valves primordia (Fig. 1h). The right and left aortic and pulmonary valve cushions develop from the unfused margins of the CRs, whereas the posterior (non-coronary) aortic and anterior (pulmonary) valve cushions develop from the ICs. The valve cushions differentiate in the adult leaflets and sinuses of the semilunar valves.

### Mechanism of fusion of the conal ridges

**Fig. 2:**
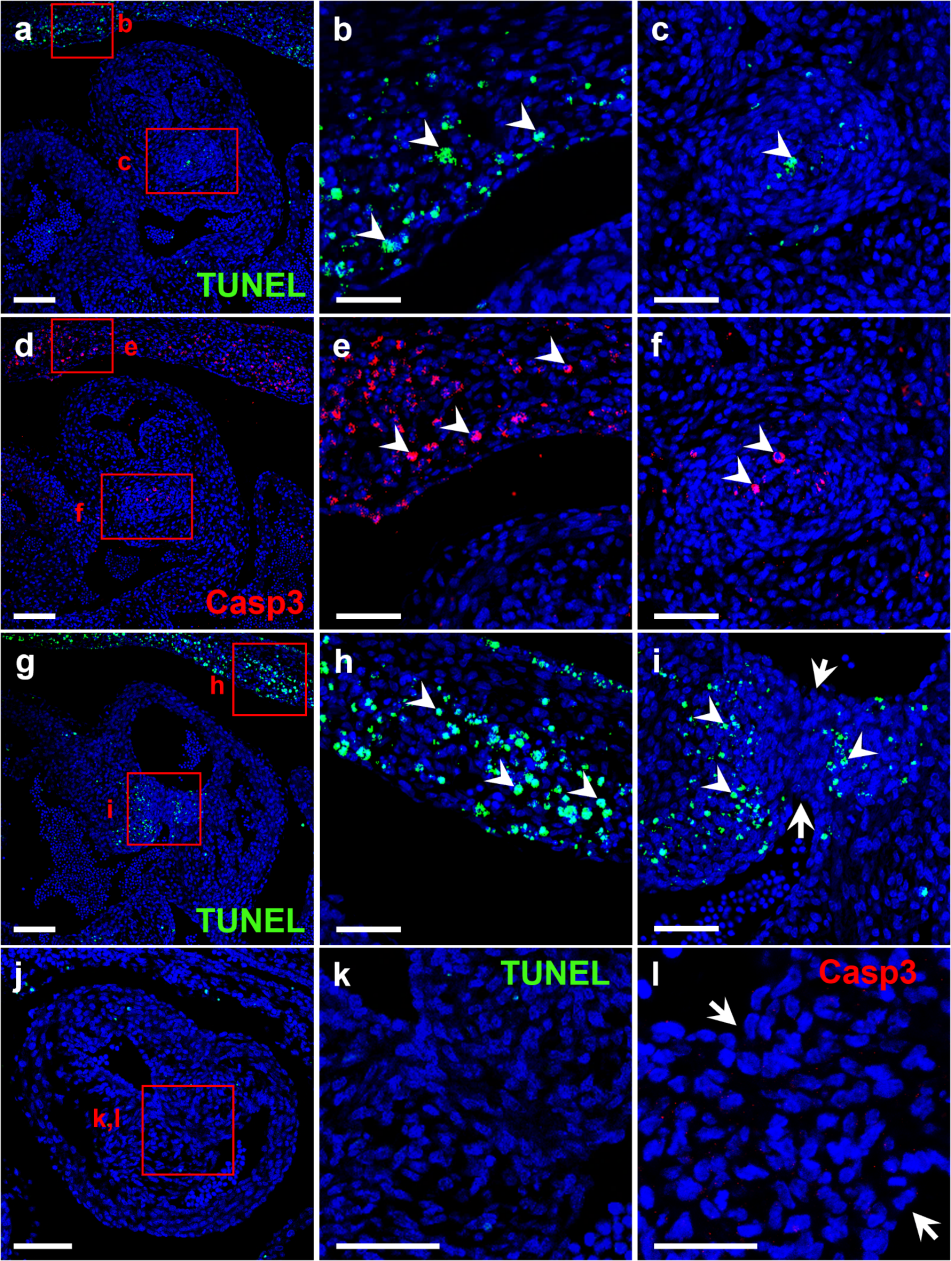
Transverse sections of the OFT of ED12 (**a**–**i**) and ED11 (**j**–**l**) hamster embryos with TAV from T and control strains (**a**–**c**) and BAV from T strain (**d**–**l**), stained with the TUNEL assay (green;** a**–**c**,** g**–**k**) or with caspase antibodies (red;** d**–**l**). Nuclei were stained with DAPI (blue). The red rectangles show magnifications of the thoracic wall (**b**,**e**), the CS (**c**,**f**) and the area of fusion of the CRs (**i**,**l**). The fusion area shown in (**j**–**l**) corresponds to the H-E stained section in Fig. 1e,m, and that shown in (**g**,**i**) corresponds to Fig. 1i. Arrows in i and l delimit the fusion area. Arrowheads point to apoptotic cells. Scale bar for insets: 100 μm. Scale bar for magnifications: 50 μm.

Inspection of transverse serial sections of the OFT of ED11 and ED12 embryos from both the control and the T strain revealed TUNEL^+^ and caspase 3^+^ cells in the thoracic wall (Fig. 2a,b,d, e,g, h), the mesenchymal cells of the core of the proximal CRs (Fig. 2g,i), and in the CS (Fig. 2a,c,d,f) of ED12 embryos. The amount and location of positive signals were similar in both strains (Fig. 2a,c,d,f and Supplemental Fig. 1a). However, no positive signals were found in the fusion area of the CRs in ED11 (Fig. 2j-l) and ED12 (Fig. 2g,i) embryos.

**Fig. 3:**
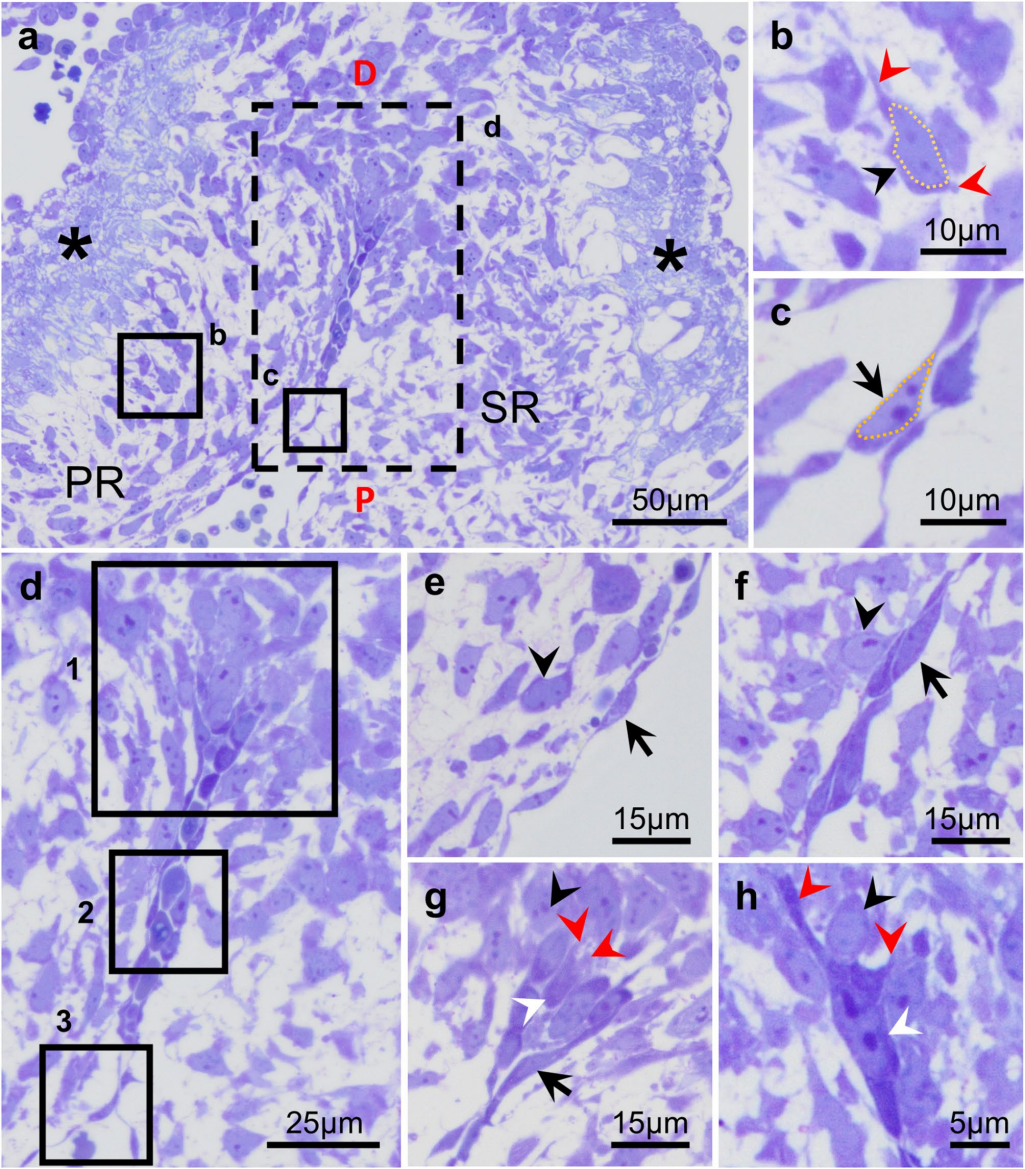
Frontal semithin sections of the middle OFT of ED11 hamster embryos stained with Toluidine Blue-Basic Fuchsin, showing the fusion area in detail. Each CR was composed of mesenchymal cells covered by a monolayer of endocardial cells (**a**–**c**). In the disto-proximal axis of the endocardium covering the CRs (**d**), three different areas could be distinguished (1–3). In the proximal region (3), septal and parietal CRs were separated, and endocardial cells presented a conventional endothelial morphology (**e**). In the middle region (2), the two opposite endocardial linings in contact exhibited a similar morphology to that described in e (**f**). In the most distal region (1), corresponding to the fusion area, endocardial cells from both CR were also in contact, but showed an intermediate phenotype between endothelial and mesenchymal cells. The intermediate phenotype was characterized by an endothelial cell appearance with features similar to those found in mesenchymal cells (**g**,**h**). D: distal, P: proximal, PR: parietal ridge, SR: septal ridge. Asterisk: conal myocardium. Black arrows point to endothelial cells, black arrowheads to mesenchymal cells and white arrowheads to intermediate phenotype. Red arrowheads indicate lamellipodia and filopodia.

In order to assess EMT process during the fusion of the CRs (Fig. 3), we first performed a fine morphological study using frontal semithin sections of the OFT to analyze the fusion area of ED11 embryos. Each CR was composed of mesenchymal cells covered by a monolayer of endocardial cells (Fig. 3a). Mesenchymal cells in the core of the CRs were featured by a small cell body, few long and thin membrane projections (i.e., filopodia and lamellipodia), and a large, round nucleus with disseminated chromatin (Fig. 3b). Most endocardial cells showed a flat morphology with a prominent, flattened, elliptical nucleus that occupied most of the cell body (Fig. 3c). Three consecutive disto-proximal areas could be distinguished in the endocardium covering the CRs (Fig. 3d). In the proximal area (Fig. 3e), septal and parietal CRs were separated, and endocardial cells presented the typical endothelial morphology described above. In the middle area (Fig. 3f), the two opposite endocardial linings were in contact, so that endocardial cells did not face the conal lumen, but exhibited a similar morphology to that described earlier. In the most distal area (Fig. 3g), corresponding to the fusion area, endocardial cells from one CR were also in contact with endocardial cells from the opposite ridge, but showed a distinct morphology. Three types of cells could be described in this location: (1) cells with a normal endothelial morphology (black arrows in Fig. 3c,e,f); (2) cells with mesenchymal morphology (black arrowheads in Fig. 3b,f-h); and (3) cells with both endothelial and mesenchymal features (white arrowheads in Fig. 3g,h). This intermediate phenotype was characterized by an endothelial cell appearance with moderately flattened morphology and hypertrophied nucleus, together with extensions of the plasmatic membrane, compatible with filopodia and lamellipodia, similar to those found in mesenchymal cells (Fig. 3g,h). Cells with mixed phenotype were predominantly present in the central portion of the fusion area (Fig. 1e,m,n, Supplemental Fig. 2).

**Fig. 4:**
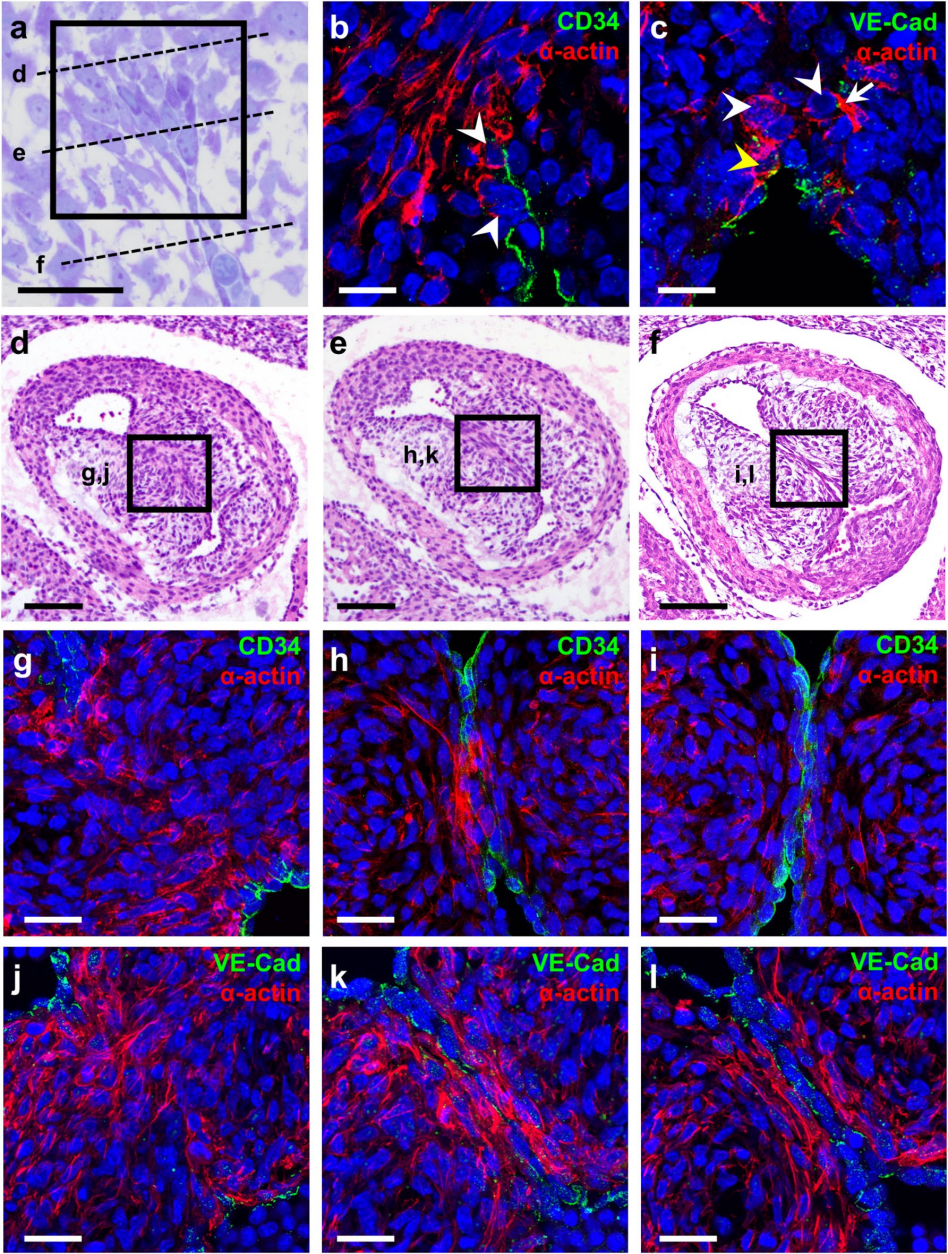
Frontal (**a**–**c**) and transverse (**d**–**l**) sections of the middle OFT of ED11 hamster embryos. (**a**) Frontal semithin section of the fusion area stained with Toluidine Blue-Basic Fuchsin. Dashed lines mark the plane of sections shown in (**d**–**f**). (**b**,**c**,**g**–**l**) Histological sections immunostained with CD34 or VE-Cadherin (green) and α-actin (red). White arrowheads point to double stained cells, yellow arrowheads to intracellular VE-Cadherin, and white arrows to forming lamellipodia and filopodia. Nuclei are stained with DAPI (blue). (**d**–**f**) Overviews of the OFTs stained with hematoxylin-eosin showing the fusion process transversally. Black rectangles in **d**–**f** mark magnified areas shown in **g**,**j**; **h**,**k** and **i**,**l**, respectively. Scale bar for insets: 100 μm. Scale bar for magnifications: 25 μm.

We also conducted double immunofluorescence with endocardial (CD34 and VE-Cadherin) and migration (α-actin) markers in frontal (Fig. 4a-c) and transverse (Fig. 4d-l) histological sections of the OFT. Lining endocardial cells showed CD34^+^ signals in the outer cell membrane (Fig. 4b,i) and VE-Cadherin^+^ punctate intercellular signals (Fig. 4c,l). Endothelial markers were absent from the area of the CR where the fusion had already taken place (Fig. 4g,j), indicating the disappearance of the endothelial lining associated with CR fusion. α-actin^+^ mesenchymal cells were particularly frequent in the most distal region of the *conus*, just above the area of fusion of the CRs (Fig. 4b,c), along the line of fusion (Fig. 4g,j) and in the central portion of coalesced CRs (Fig. 4h,k). In the fusion area we constantly found some endocardial cells that showed CD34, α-actin and VE-Cadherin positive signals (Fig. 4b,c,h,k). These cells were in close contact with the before mentioned α-actin^+^ mesenchymal cells, and were located where cells with an intermediate morphological phenotype were previously detected (Fig. 4a-c). In addition, some of the VE-Cadherin^+^ cells in the fusion area showed delocalization of the membrane punctuate signal, shifting its location to the cellular cytoplasm (Fig. 4c).

## Fusion of the conal ridges during bicuspid aortic valve formation

**Fig. 5:**
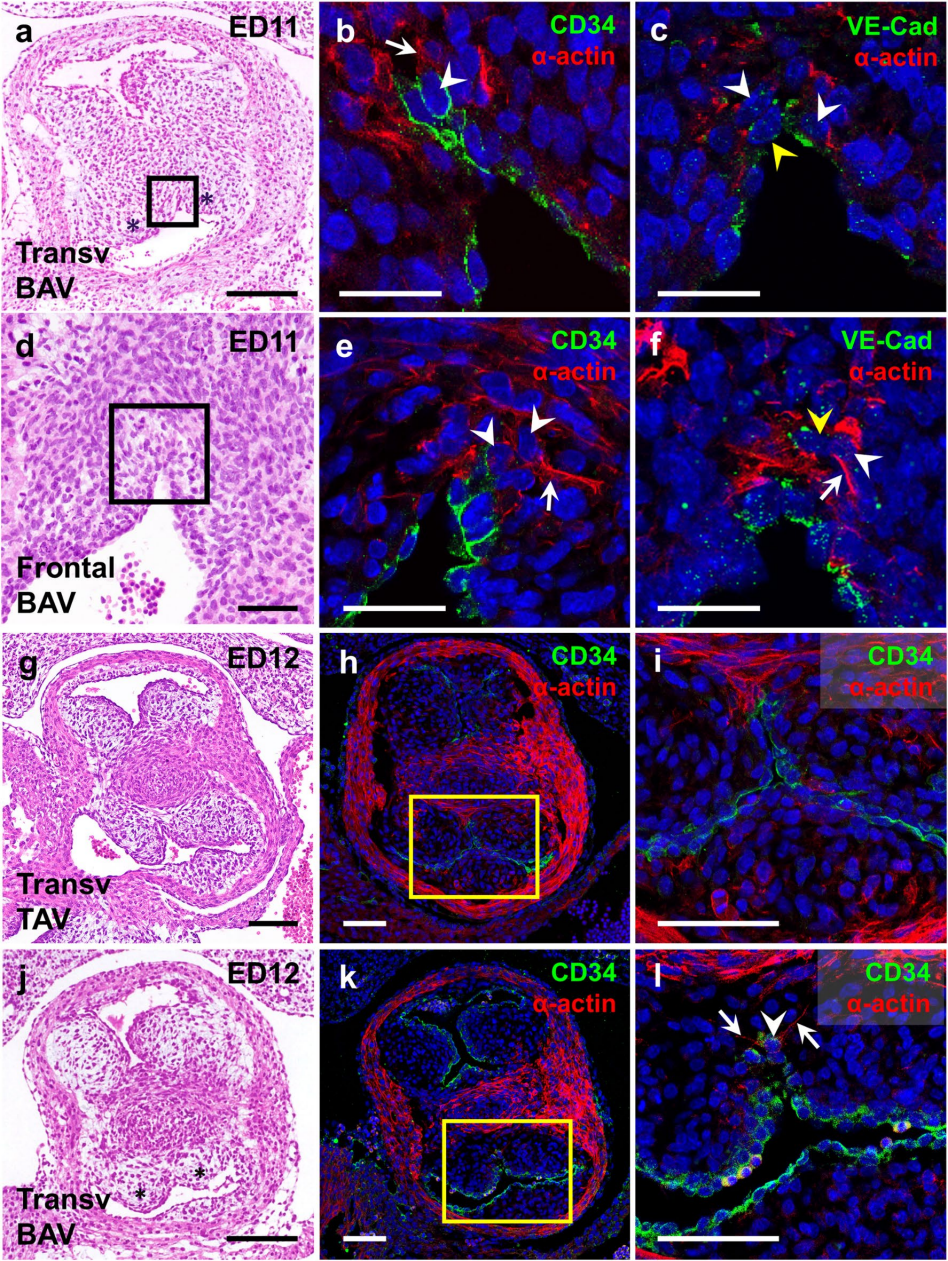
Transverse (**a**–**c**,** g**–**l**) and frontal (**d**–**f**) sections of the middle region of the OFT of ED11 (**a**–**f**) and ED12 (**g**–**l**) hamster embryos. Normal (TAV) and BAV embryos are shown in (**g**-**i**) and (**a**–**f**), (**j**–**l**), respectively. Overviews in (**a**,**d**,**g**,**j**) are stained with hematoxylin-eosin. Sections immunostained with CD34 or VE-Cadherin (green) and α-actin (red) are shown in (**b**,**c**,**e**,**f**,**h**,**i**,**k**,**l**. Nuclei were stained with DAPI (blue). Black rectangles in a and d indicate magnified areas shown in **b**,**c** and **e**,**f** respectively. Yellow rectangles in h and k indicate magnified areas shown in **i** and **l**, respectively. White arrowheads point to double stained cells, yellow arrowheads to intracellular VE-Cadherin and white arrows to forming lamellipodia and filopodia. Asterisks in **a** and **j** indicate fused cushions in a BAV embryo. Scale bar for insets: 100 μm. Scale bar for magnifications: 50 μm.

A developing BAV was detected in 15 out of 40 (37.5%) ED11 and ED12 embryos of the T strain. In these embryos, an excessive fusion of the CRs (ED11, Fig. 5a) or fused right and left aortic valve cushions (ED12, Fig. 5j) were observed.

In ED11 embryos developing BAV, the fusion of the CRs was not restricted to their central portions but extended to the posterior margins of the CRs (Fig. 5a). Double immunofluorescence with CD34 or VE-Cadherin and α-actin in transverse (Fig. 5b,c) and frontal (Fig. 5e,f) sections of the OFT revealed the presence of CD34^+^; α-actin^+^ and VE-Cadherin^+^; α-actin^+^ cells in the region of CRs affected by extra-fusion. These cells were present in the fusion area, adjacent to the contacting endocardia in the extra-fusion region (Fig. 5b,c). As described in embryos developing TAV, there were VE-Cadherin^+^ cells in which the signal was found in the cellular cytoplasm (Fig. 4c). The analysis of frontal sections of the fusion area (Fig. 4e,f) revealed α-actin^+^ mesenchymal cells located in the most distal portion, CD34^+^; α-actin^−^ and VE-Cadherin^+^; α-actin^−^ endocardial cells in the most proximal region, and double-labelled cells in the intermediate zone. This cellular distribution pattern was very similar to that described in normal embryos (compare Fig. 4a-c with Fig. 5d-f), and the number of doubled-labelled cells was significantly higher in the fusion area of BAV embryos as compared with embryos with a normal valve from the T (p-value: 1,36E-07) and control (p-value: 1,46E-07) strains (Supplemental Fig. 1b).

After fusion of the CRs (ED12), the CS develops in the center of the fused area, and the aortic and pulmonary valve cushions become delineated in the OFT (Fig. 5g). In embryos developing TAV, the right and left aortic valve cushions were delimited by the OFT lumen, the CS and the conal myocardium, while the posterior or non-coronary valve cushion was delimited by the OFT lumen and the conal myocardium (Fig. 5g). Double immunofluorescence for CD34 and α-actin showed that α-actin^+^ signals were mainly found in the CS and the conal myocardium, whereas endocardial cells were positive for CD34 endothelial marker (Fig. 5h). The endocardium that delimited the left and right aortic valve cushions reached the CS, demarcating two separated and well-defined cushions (Fig. 5i). No CD34^+^; α-actin^+^ cells were detected near the endocardium (Fig. 5i). By contrast, in embryos developing BAV, the extended fusion of the posterior margins of the CRs had led to fusion of the right and left aortic valve cushions (Fig. 5j). CD34^+^ endocardial cells lined the left and right aortic valve cushions, but the endocardium did not reach the CS, showing two fused valve cushions (Fig. 5j,k). However, some cells of the fused region of the cushions located near the endocardium exhibited positive double signals for CD34 and α-actin (Fig. 5l).

## Discussion

The division of the embryonic common OFT, usually referred to as OFT septation, is a key developmental process for the conversion of a single to a double circulatory system^1–4,7^. Defects in OFT septation lead to multiple cardiac abnormalities, the sum of which constitute a wide majority of the human congenital cardiac malformations^4,5,28^. Cardiac OFT septation is an evolutionary conserved embryonic mechanism in birds and mammals^6,28^, involving the fusion of the CRs to form the mesenchymal CS, and the aortic and pulmonary valve primordia^2–4,7^.

Conal septation in the Syrian hamster occurs between stages ED11 (fusion of the CRs) and ED12 (formation of the CS). The fusion of the CRs entails disappearance of the endocardium covering the two opposite CRs after contact^27^. Three fusion mechanisms have been proposed to operate during embryonic development in different morphogenetic processes, including the fusion of the CRs: epithelial adhesion, epithelial apoptosis and epithelial-mesenchymal transition (EMT)^27^. These mechanisms are typically involved in fusion events such as neural tube formation or palate development. The first mechanism requires the expression of adhesion molecules and the maintenance of the identity of cells in contact, which occurs during early stages of neural tube formation^29,30^. In the other two mechanisms, epithelial cells covering the fusing structures disappear by cell death (apoptosis) or by transforming into mesenchymal cells (EMT), both cellular mechanisms underlying the palatal shelf fusion^31^. Since the endothelium that constitutes the endocardium is a specialized squamous epithelial tissue^32^, the mechanisms described can be extrapolated to the endocardium. Given that endocardial cells disappear during fusion of CRs^27^, we selected apoptosis and EMT as possible candidate mechanisms to be tested.

**Fig. 6:**
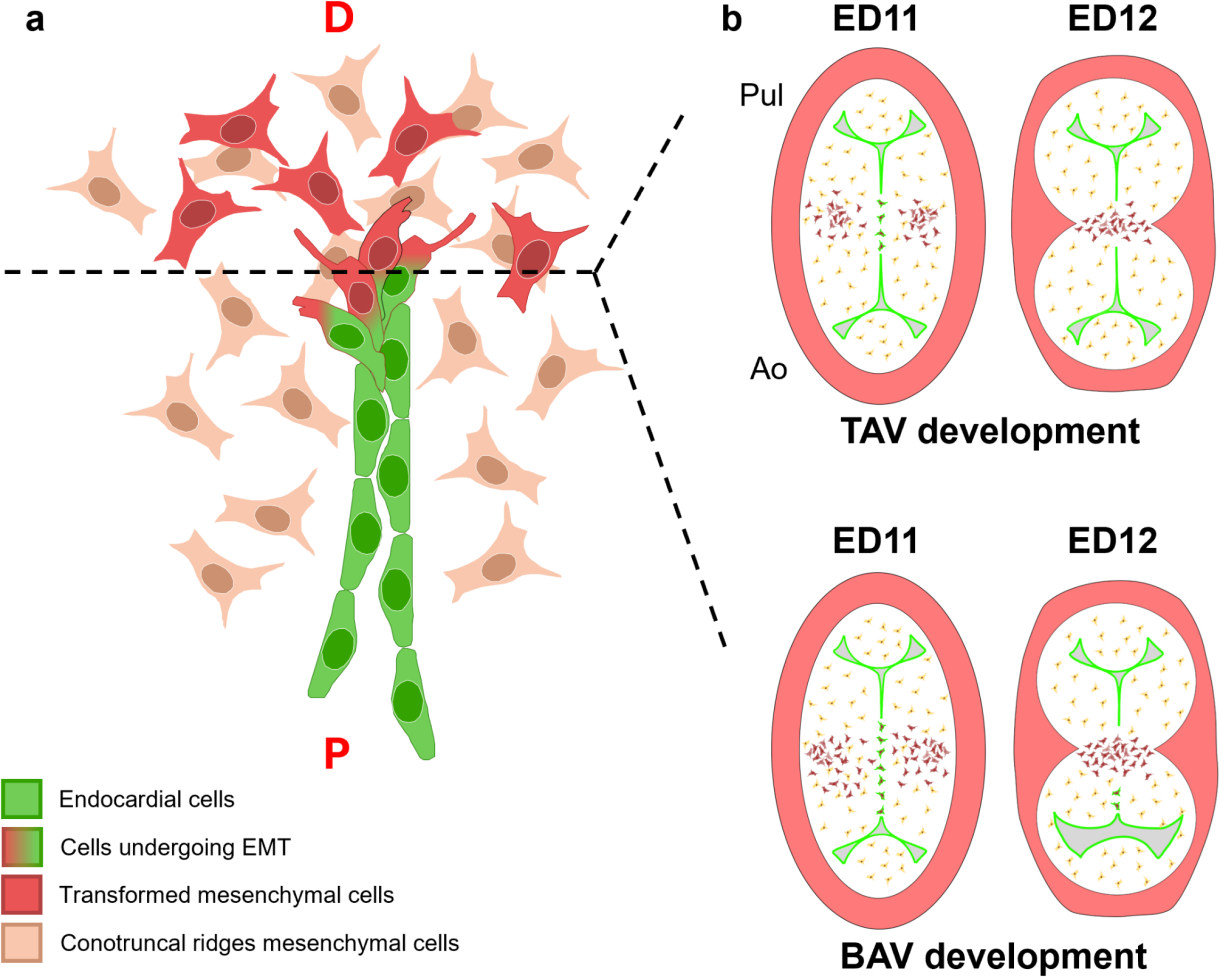
Schematic representation of EMT process during the fusion of CRs in frontal (**a**) and transverse (**b**) views of the OFT during normal (TAV) and bicuspid aortic valve (BAV) development. (**a**) The EMT process occurs sequentially along the disto-proximal axis of the OFT. Proximal (P) endocardial cells (green) are quiescent. Mid-portion endocardial cells (in red and green) are undergoing EMT, showing an endocardial-mesenchyme mixed morphology. Distal (D) mesenchymal cells (in red) are transformed from endocardial cells. Brown cells are mesenchymal cells that form the core of CRs. (**b**) Transforming endocardial cells involved in the fusion event are restricted to the center of the CRs (green cells) of ED11 TAV embryos, whereas in BAV embryos the number of transforming cells increase in the posterior (aortic) region of the OFT, continuing at ED12. Therefore, spatial and temporal alteration of the EMT process underlies the development of BAV. Pul: pulmonary artery, Ao: aorta artery.

Apoptosis was not found in the fusion area of CRs of hamster embryos (Fig. 3). However, we identified apoptotic cells in other embryonic regions, such as the CS, the mesenchymal cells from the core of CRs, and the thoracic wall, in which the presence of apoptosis had been previously described^33–35^. Regarding EMT, fine morphological analysis and colocalization of endocardial and migration markers determined the presence of three different cell types in the fusion region (Figs. 3 and 4): (1) CD34^+^;VE-Cadherin^+^ cells in the proximal region of the fusion area, showing a typical endothelial morphology^36,37^; (2) α-actin^+^ cells in the distal area to the fusion zone, which presented a typical mesenchymal morphology^37,38^; and (3) CD34^+^;VE-Cadherin^+^;α-actin^+^ cells with a mixed phenotype between endothelial and mesenchymal cells, located between the two cell types mentioned above. This intermediate phenotype includes morphological features previously ascribed to EMT^38–40^ (Fig. 6). Additionally, we found delocalization of the VE-Cadherin punctuate signaling, shifting to intracellular in double-labelled cells (Figs. 4c and 5c). This change of protein distribution has been described in EMT cells as a result of endothelial detachment^41^.

These findings clearly indicate that EMT, and not apoptosis, is the cellular mechanism underlying CR fusion. The endocardial cells in the central region of the opposing CRs disappear by transforming into mesenchymal cells, morphologically indistinguishable from the rest of mesenchyme in the cushions core, and leading to morphological continuity between the CRs (Fig. 6). The distribution of each cell type in the fusion area allows establishing a sequential pattern of the EMT process (Figs. 1l-m, 3 and 4). It can be inferred that the most proximal CD34^+^; VE-Cadherin^+^; α-actin^−^ endothelial cells have not yet initiated EMT; cells with intermediate phenotypes and positive signals for the three markers are endothelial cells transforming into mesenchymal cells; and in the most distal α-actin^+^ mesenchymal cells, the transition event is ending (Fig. 4). Thus, this pattern reflects a disto-proximal sequence of cellular events consistent with the EMT mechanism (Fig. 6). Likewise, the whole morphogenetic process of conal septation follows the EMT disto-proximal sequence described.

Two cellular mechanisms have been ascribed to the EMT process in the literature: partial EMT and complete EMT^42^. In the former, transformation may affect only a few cells within the epithelium or may occur through stages, in such a way that the epithelium remains intact during the entire process, being the transformed epithelial cells replaced by neighbors. In the latter mechanism all cells in the epithelium undergo transformation simultaneously, with a complete disintegration of the epithelial cover that transforms into mesenchyme. Both EMT mechanisms have been previously described in processes involved in embryonic development, such as delamination of cranial neural crest cells and sclerotome and dermatome development, respectively^43,44^. The results obtained in this study allow us to suggest that the fusion of CRs is based on a complete EMT mechanism that sequentially affects the underlying endocardial cells located in the center of the CRs. This would explain the presence of cells with different phenotypes in the fusion area, whereas endothelial cells lateral to the fusion area remain unaffected, constituting the endothelial cover of the valve cushions (Supplemental Fig. 2, Fig. 6). The specific location of the EMT process in the center of the CRs might be the consequence of the specific and localized induction by CNC cells.

R-L BAV in hamsters results from the excessive fusion of the CRs, leading to the formation of two instead of three aortic valve cushions^9,20^. We reasoned that the excessive fusion of the CRs might be caused by an extension of the EMT process to their posterior margins, affecting the adjacent areas of the presumptive right and left aortic valve cushions. To test this hypothesis, we examined ED11 and ED12 hamster embryos from the T strain, with relatively high incidence of R-L BAV (~40%). Fifteen out of 40 (37.5%) embryos exhibited a developing BAV. Immunostainings of BAV embryos at ED11 showing an excessive fusion of CRs (Fig. 5a), revealed the existence of CD34^+^; VE-Cadherin^+^; α-actin^−^ signals in the lining endocardium, CD34^−^; VE-Cadherin^−^; α-actin^+^ cells in the mesenchyme core of the CRs, and CD34^+^; VE-Cadherin^+^; α-actin^+^ cells specifically in the region of CRs affected by extra-fusion (Fig. 5b,c,e,f), demonstrating a distribution pattern very similar to that found in normal embryos. Double-labelled cells were significantly more abundant in the complete fusion area of BAV embryos as compared with embryos with TAV (Supplemental Fig. 1b). These findings suggest that EMT underlies the extra-fusion of CRs in BAV embryos, and reinforce the hypothesis proposed above of a fusion event based on a sequential and complete EMT mechanism.

At ED12, TAV specimens presented a CD34^+^ endocardium that reached the CS and delimited two separated and well-defined left and right aortic valve cushions. However, in BAV embryos from the T strain, CD34^+^ lining endocardium did not reach the CS, allowing the continuity between the mesenchymal masses of the left and right aortic valve cushions (Fig. 5i,l). Some CD34^+^; α-actin^+^ signals were constantly detected in cells adjacent to the endocardium of the contacting valve cushions. We interpret that these double-labelled cells are undergoing EMT. These findings show for the first time that the fusion of CRs can continue after the formation of the CS.

We propose that during the formation of R-L BAVs, the number of endocardial cells involved in the EMT process is altered, resulting in a spatial (i.e., spreading of the fusion towards the posterior limits of the CRs) and temporal (i.e., after de CS formation) extension of the fusion of the CRs (Fig. 6). The alteration of EMT underlying BAV formation is supported by several genetically modified mouse strains (reviewed in^9^), in which defective expression of genes known to regulate EMT during OFT development, such as *eNOS*, *Brg1*,* Ift88* and *Npr2*^20,45–47^, leads to BAV as a consequence of dysregulation of EMT that affects cushion formation. In addition, a recent study on BAV-associated aortopathy demonstrated overexpression of EMT genes related to endocardial cushion formation in the aorta of affected patients^48^, suggesting a common embryologic defect that disturbs the development of the aortic wall and the aortic valve.

The EMT dysregulation hypothesis seems to match with the fact that R-L BAV originates from an abnormal distribution of CNC cells^20^. It has been shown that the CNC is essential for proper OFT septation and formation of the arterial trunks and semilunar valves. CNC cells form the so-called aortic-pulmonary septation complex (Fig. 1a). Migration of this complex takes place disto-proximally through the CRs and is responsible for the fusion event^2,3,8,49–51^. We propose that CNC cells of the aortic-pulmonary septation complex induce EMT of contacting endocardial cells located close to the forming CS (complete EMT), leading to a disto-proximal sequential fusion of CRs. The altered migration of CNC cells along the OFT would entail an extension of the endocardial territory affected by the EMT induction, increasing the number of activated endocardial cells that results in an excessive fusion of the CRs. This mechanism is consistent with the study of Plein et al., 2015^51^, who demonstrated that migration of CNC and EMT are interconnected morphogenetic processes. They found that a specific population of CNC cells induces EMT of the lining endocardium in early stages of cardiogenesis, promoting the cellularization of the endocardial cushions. Endocardial cells in turn regulate CNC migration through the endocardial cushion mesenchyme to form the two cell condensations of the aortic-pulmonary septation complex. The regulation of this event implies the establishment of a crosstalk between the CNC and the endocardium. Class 3 semaphorin (SEMA3C), an autocrine factor secreted by CNC cells, and neuropilin 1 (NRP1), a SEMA3C receptor located in the endocardial cell membrane, are key molecular elements involved in this crosstalk system^51^. Considering that CNC-derived SEMA3C and endocardial NRP1 are required for both, endocardial EMT and CNC migration, it seems plausible that this intercellular communication system is also responsible for the whole process of conal septation, including fusion of CRs by EMT and formation of the CS by migrating CNC cells from the cell condensations. Indeed, this pathway has been demonstrated to be required specifically for the migration of CNC cells to the fusion area of CRs^51^. Another molecular pathway possibly involved is DLL4-JAG1-NOTCH1, which is required in the endocardium to ensure proper valve morphogenesis and cardiac septation through regulation of EMT^52,53^. The alteration of this signaling pathway gives rise to different types of BAV, including R-L BAV^52^. Given that NOTCH signaling is necessary for CNC patterning in the OFT^54^, MacGrogan et al. (2016)^52^ proposed that the endocardium is likely responsible for the regulation of the CNC migration through the NOTCH pathway. It would be of interest to delve into whether any of the signaling pathways described are also involved in the EMT process underlying the fusion of CRs.

### Limitations of the study

The only spontaneous animal model of BAV disease, consisting of an inbred strain of Syrian hamsters^13,23,24^, was used to conduct this study. The current unavailability of cell lineage tracing strategies for this species has hampered the acquisition of direct experimental evidence for involvement of EMT in the fusion mechanism of the CRs. The experimental strategy used to overcome this limitation was based on the accumulation of indirect experimental evidence, firstly by eliminating alternative cellular mechanisms (apoptosis), and, secondly by confirming the most promising candidate mechanism with different experimental approaches (i.e., expression analysis of EMT molecular markers combined with the identification of fine cellular morphological features associated with the EMT process, according to current guidelines^40^). Cell lineage tracing analysis in the context of this investigation has not been addressed in any transgenic mouse model, which would be of interest (1) to confirm that the fusion process described in hamsters also occurs in mice, and (2) to reinforce the extrapolation of the mechanism to humans.

## Methods

### Animals

Animals were handled in accordance with the European guidelines (Directive 2010/63/EU) and Spanish regulations (R.D. 53/2013; B.O.E. 01.02.2013) for the protection of experimental animals.

A total of 108 hamster embryos aged between 11 and 12 days postcoitum (ED11 and ED12) were analyzed. Sixty-one of these embryos belonged to an inbred strain with around 40% incidence of non-syndromic isolated BAV type A (R-L fusion type, including fused BAV, 2 sinus BAV and partial fusion BAV, according to^17^). The T strain was developed and is maintained by the research group in the University of Malaga. The characteristics of this strain (T strain) have been previously reported^9,13,20,22–25^. The remaining 47 embryos belonged to an outbred strain (RjHan: AURA; Janvier, France; control strain) with null incidence of BAV, which has been previously used as a control for the T strain^25^.

The embryonic stage was determined by counting the days from the moment of the copula, which was directly visualized and designated as stage ED0. The embryos were harvested at the required stage by laparotomy and hysterectomy from the pregnant female and sacrificed by immersion in ice-cold phosphate buffer saline (PBS) 0.02 M.

### Histochemistry

ED11 and ED12 embryos (T strain: *n* = 56; control strain: *n* = 38) were fixed by immersion in 4% paraformaldehyde diluted in PBS overnight. Then, the embryos were dehydrated in a graded series of ethanol and embedded in paraffin (Histosec, Merck, Germany). The embryos were orientated to obtain eight µm thick transverse or frontal serial sections (Fig. 1b) of the heart in a Leitz 1512 microtome. Some of these sections were dewaxed, hydrated, and stained with Delafield haematoxylin-eosin. Samples were visualized and photographed with a Leica DMSL light microscope equipped with a Leica DFC 500 camera or in an Olympus VS120 automated slide scanner.

### TUNEL assay

The terminal deoxynucleotidyl transferase-mediated deoxyuridine triphosphate nick end labeling (TUNEL) assay was used to detect apoptosis in a total of 29 ED11 and ED12 embryos with TAV (n = 12; ED11: 7; ED12: 5) and BAV (n = 4; ED11: 2; ED12: 2) from T strain, and TAV embryos from the control strain (n = 13; ED11: 8; ED12: 5). A commercial kit (Roche, Switzerland) was employed following the manufacturer’s instructions. Omission of the transferase enzyme was used as a negative control. Finally, the sections were stained with 4’,6-diamidino-2-phenylindole dihydrochloride (DAPI, Sigma-Aldrich, USA) diluted 1/1000 in PBS to verify the nuclear location of the TUNEL signals. The sections were observed and photographed with a Leica Stellaris confocal microscope.

### Single and double immunofluorescence

Sections from 75 ED11 and ED12 embryos belonging to the T strain (*n* = 40) with TAV (ED11: 12; ED12: 13) and BAV (ED11: 8; ED12: 7), and the control strain with TAV (*n* = 25; ED11: 14; ED12: 11) were deparaffinated and washed in Tris-PBS (TPBS, pH 7.8). Non-specific binding sites were saturated for 1 h with 10% bovine serum, 1% bovine serum albumin and 0.1% Triton X-100 in TPBS (BST). The Avidin-Biotin blocking kit from Vector was used to block endogenous biotin. Then, sections were incubated with the primary antibody (Anti-Caspase 3, Anti-CD34, Anti-VE-Cadherin; details are provided in Supplemental Table 1) at 4 °C overnight, rinsed in TPBS and incubated with an anti-rabbit IgG biotin conjugate (Sigma-Aldrich, USA, REF: B8895) for 1.5 h at RT. After washing with TPBS, sections were incubated with FITC- or Cy3-conjugated extravidin (Sigma-Aldrich, USA) diluted 1/250 in TPBS for 1 h at RT. Negative control samples were incubated with BST in the absence of the primary antibody. For CD34 and VE-Cadherin immunofluorescence, sodium citrate buffer (10mM Sodium Citrate, 0.05% Tween 20, pH 6.0) and EDTA buffer (1mM EDTA, 0.05% Tween 20, pH 8.0) were respectively required for antigen retrieval (boiling in microwave for 15 min), prior to blocking the non-specific bindings. After retrieval, samples were cooled at RT and rinsed with TPBS. For double immunofluorescence, Cy3-conjugated anti-α-actin smooth muscle (mouse monoclonal, Sigma-Aldrich, USA, REF: C6198) diluted 1/300 in BST was incubated for 1.5 h at RT. Nuclei were counterstained with DAPI diluted 1:1000 in TPBS. Slides were observed and photographed with a Leica Stellaris confocal microscope. Sections were analyzed in a Z-stack (2 μm/plane).

### Quantification of apoptotic and EMT+ cells

For quantification of cells undergoing apoptosis and EMT, we used IF for caspase 3 and double immunofluorescence with CD34 and α-actin, respectively. The analysis was assesed in consecutive sections (x40) of the complete fusion area of the CRs (i.e., from areas of complete fusion to visible contacting endocardia) of control embryos and embryos belonging to the T strain with TAV and BAV at ED12 for apoptosis and ED11 for EMT markers. Differences between two groups were analyzed using the Student’s t-test and were considered statistically significant when p-value < 0.05. The statistical analyses were performed with the SPSS 27.0 software. All graphics and data show the mean value and the standard error of the mean.

### Semithin sections

Eighteen ED11 embryos (T strain: *n* = 9; control strain: *n* = 9) were immersion fixed with a mix of 1% paraformaldehyde, 2% glutaraldehyde and 0.05 M cacodylate buffer for 1 h, washed in cacodylate buffer for 30 min and post-fixed by immersion in 1% osmium tetroxide diluted in 0.05 M cacodylate buffer for 1.5 h. Then, samples were rinsed in distilled water several times until complete removal of fixative. After that, they were dehydrated in graded acetone concentrations, washed in propylene oxide (Sigma-Aldrich, USA), and embedded in Araldite epoxy resin (Electron Microscopy Sciences, Hatfield, PA, US). Embryos were oriented to provide frontal sections of the cardiac OFT (Fig. 1b). One µm serial sections were obtained in a Leica UC7/FC7 ultramicrotome, stained with Toluidine Blue-Basic Fuchsin (Sigma-Aldrich, USA) and photographed in an Olympus VS120 automated slide scanner.

## Electronic supplementary material

Below is the link to the electronic supplementary material.

Supplementary Material 1

Supplementary Material 2

Supplementary Material 3

## Data Availability

The data generated and analyzed in this study are available in this article in the main manuscript and supplementary file.
