# Supplementary figures and images for: Endocardial-to-mesenchymal transition underlies cardiac outflow tract septation and bicuspid aortic valve formation in the Syrian hamster model
Source: Sci Rep. 2025 Mar 12;15:8583. doi: 10.1038/s41598-025-91454-6 (PMC11903957; doi:10.1038/s41598-025-91454-6)

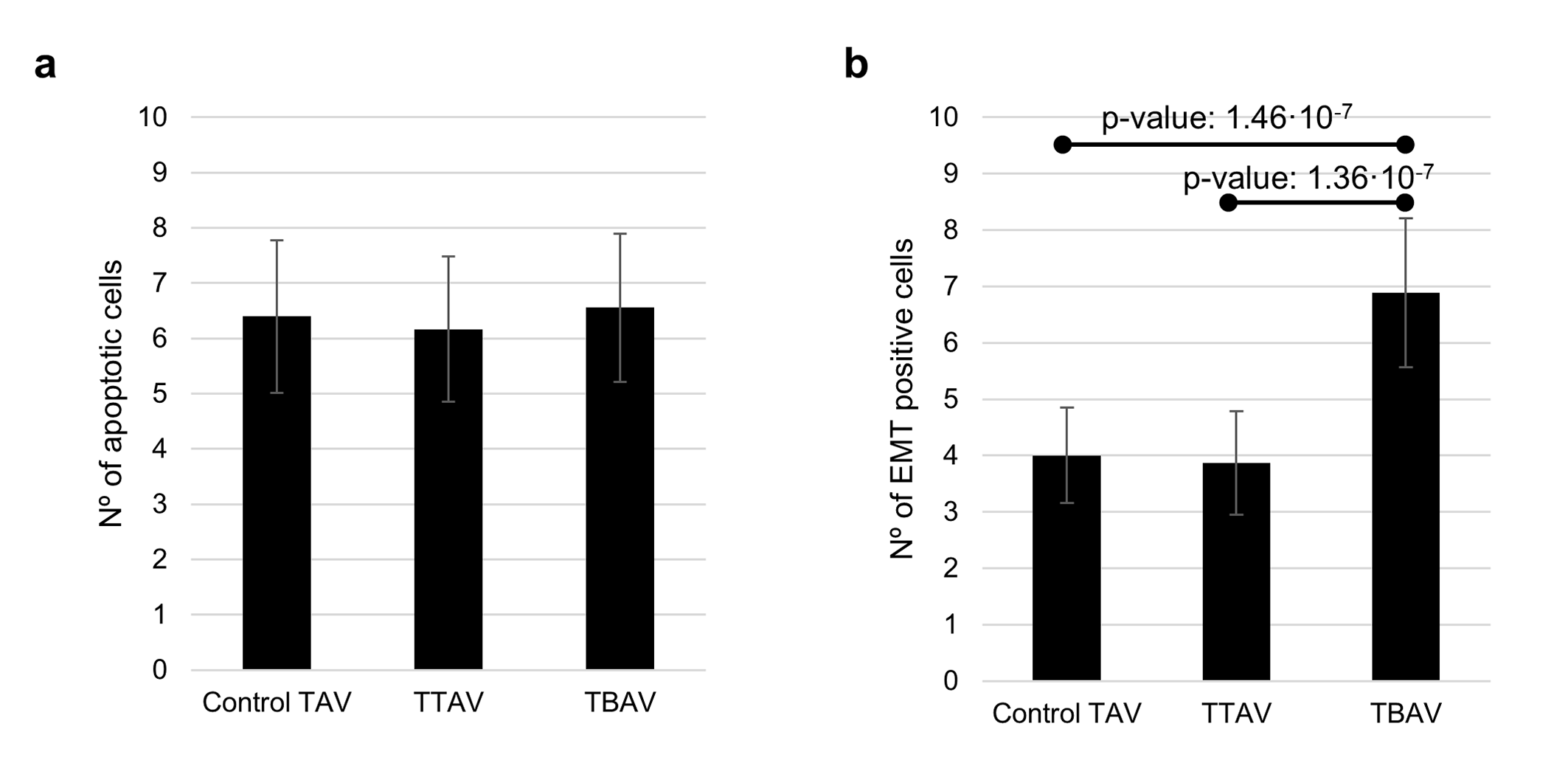

Supplement: Supplementary file 1 — Supplementary Material 1 [file 41598_2025_91454_MOESM1_ESM.tif]

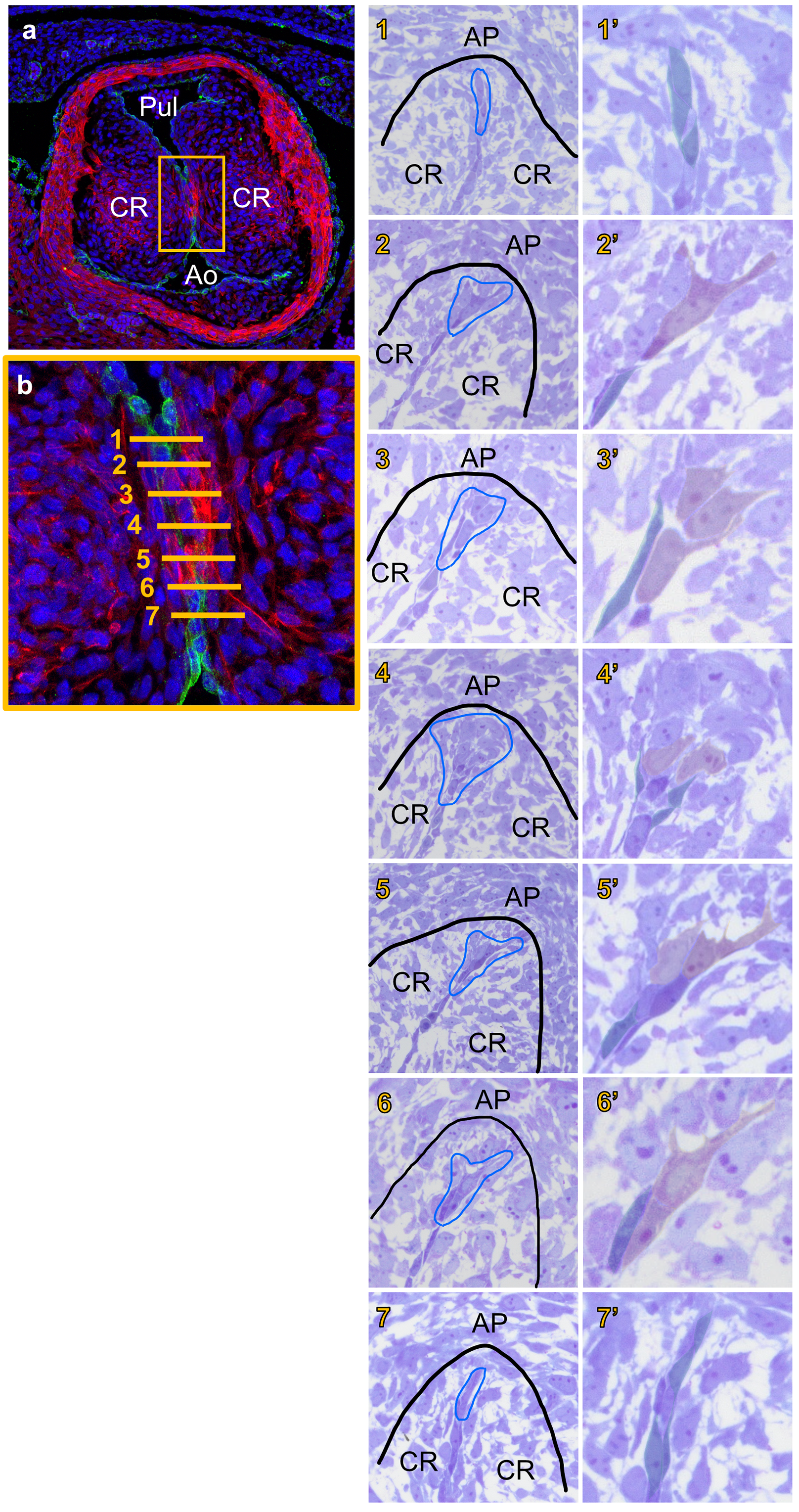

Supplement: Supplementary file 2 — Supplementary Material 2 [file 41598_2025_91454_MOESM2_ESM.tif]
