# Supplementary material for: Endocardial-to-mesenchymal transition underlies cardiac outflow tract septation and bicuspid aortic valve formation in the Syrian hamster model
Source: Sci Rep. 2025 Mar 12;15:8583. doi: 10.1038/s41598-025-91454-6 (PMC11903957; doi:10.1038/s41598-025-91454-6)
Supplement: Supplementary file 3 — Supplementary Material 3 [file 41598_2025_91454_MOESM3_ESM.docx]

**Supplemental Figure 1.** Quantification of apoptotic cells in the conal septum (a) and EMT positive cells of the fusion area (b) of ED11 (b) and ED12 (a) embryos with TAV from control strain and TAV and BAV from T strain.

**Supplemental Figure 2.** Transverse (a,b) and frontal (1-7, 1’-7’) sections of the OFT of two ED11 hamster embryos.a,b: Histological section immunostained with CD34 (green) and α-actin (red) with nuclei stained with DAPI (blue). Yellow rectangle in a indicates the magnified area shown in b. Numbered yellow lines in b indicate the corresponding position of the frontal semithin sections in the transversal view. 1-7: Semithin sections stained with Toluidine Blue-Basic Fuchsin. Black lines in 1-7 delimited the aortic-pulmonary or conal septation complex (AP). The regions where the fusion takes place are delimited by blue lines and magnified in 1’-7’. No signs of EMT were found in the endocardia of the anterior (1,1’) and posterior (7,7’) margins of the contacting CRs, where only endocardial cells were found (shaded in green). However, as the sections approach the central portion of fusing CRs (2,2’,3,3’,5,5’,6,6’), the distal region of the fusion area shows a few endocardial cells initiating the EMT process (shaded in yellow). In the central portion (4,4’), there is a higher concentration of cells undergoing EMT in different stages of the process. Ao: aorta artery; CR: conal ridge; Pul: pulmonary artery.

**Supplemental Table 1.** Antibodies used for immunofluorescence.

| **Antibody** | **Host species** | **Epitope recognition** | **Working dilution** | **Commercial info** |
| --- | --- | --- | --- | --- |
| Anti-Caspase 3 | Rabbit | Polyclonal | 1/500 | Sigma-Aldrich, REF: C8487 |
| Anti-CD34 | Rabbit | Polyclonal | 1/500 | Abcam, REF: ab81289 |
| Anti-VE-Cadherin | Rabbit | Polyclonal | 1/500 | Abcam, REF: ab33168 |
| Anti-α-Smooth Muscle Actin Cy3 | Mouse | Monoclonal | 1/300 | Sigma-Aldrich, REF: C6198 |
